# Sports Massage and Blood Flow Restriction Combined with Cold Therapy Accelerate Muscle Recovery After Fatigue in Mixed Martial Arts Athletes: A Randomized Controlled Trial

**DOI:** 10.3390/jfmk10020194

**Published:** 2025-05-28

**Authors:** Robert Trybulski, Robert Roczniok, Gracjan Olaniszyn, Yaroslav Svyshch, Andryi Vovkanych, Michał Wilk

**Affiliations:** 1 Medical Department Wojciech Korfanty, Upper Silesian Academy in Katowice, 40-659 Katowice, Poland; 2 Department of Physical Therapy and Ergotherapy, Ivan Boberkyj Lviv State University of Physical Culture, 79007 Lviv, Ukraine; yarsvysch@ukr.net (Y.S.); avovkinfiz@i.ua (A.V.); 3 Institute of Sport Science, The Jerzy Kukuczka Academy of Physical Education, 40-065 Katowice, Poland; info@analizy-statystyczne.pl (R.R.) and m.wilk@awf.katowice.pl (M.W.); 4 Physiotherapy Centre “Od Nowa” Racibórz Zamkowa 4 Street, 47-400 Racibórz, Poland; gracjan.olaniszyn@wp.pl

**Keywords:** combat sports, MMA, regeneration, occlusion, manual therapy, sports medicine, ice bags

## Abstract

Objectives: The purpose of this study is to quantitatively evaluate the combined effects of sports massage, blood flow restriction (BFR), and cold therapy on quadriceps recovery in mixed martial arts (MMA) athletes following eccentric exercise, focusing on muscle biomechanical properties, pain, and strength. Methods: This randomized, single-blind clinical trial involved 36 men and women MMA-trained participants, divided into three groups: massage (n = 12) received massage, BFR/cool (n = 12) received combined BFR and cooling, and control (n = 12) received passive rest as a control. The fatigue protocol involved MMA fighters performing five sets of plyometric jumps on a 50 cm box until exhaustion, with 1-min breaks between sets. After that, the massage group received a 20-min massage overall using standardized techniques; BFR/cool underwent a 20-min alternating blood flow restriction (200 mmHg) and cooling treatment with ice bags on the quadriceps; and the final group served as the control group with passive rest and no intervention. Participants were assessed four times—before exercise, immediately after exercise, 24 h post-exercise (after two recovery sessions), and 48 h post-exercise (after four recovery sessions)—for perfusion unit (PU), muscle elasticity, pressure pain threshold (PPT), reactive strength index (RSI), and total quality recovery (TQR). Results: The statistical analysis revealed significant effects of both massage and BFR/cooling interventions across key recovery outcomes, with large effect sizes for time-related changes in RSI (*p* < 0.0001; η^2^ = 0.87), elasticity (*p* < 0.0001; η^2^ = 0.84), and PPT (*p* < 0.0001; η^2^ = 0.66). Notably, post-exercise 48 h values for RSI, elasticity, PU, and TQR were significantly improved in both the massage and BFR/cool groups compared to control (*p* < 0.05)), while no significant group differences were observed for PPT. Conclusions: The study concludes that both massage and combined blood flow restriction with cooling interventions significantly enhance post-exercise recovery—improving muscle perfusion, elasticity, reactive strength, and perceived recovery—compared to passive rest.

## 1. Introduction

Effectively addressing muscle recovery after intense physical activity is a common concern in sports training and rehabilitation [1]. Implementing successful recovery techniques is vital for athletes to maintain optimal performance levels, minimize their risk of injury, and improve overall muscle repair. In the context of mixed martial arts (MMA), athletes frequently encounter muscle pain and injuries resulting from their training and participation in official fights namely because of strikes [2]. For example, a previous study found that the mean frequency of leg kicks attempted in MMA was 4.9 ± 4.8 for split decisions, 4.2 ± 4.6 for unanimous decisions, 1.2 ± 1.9 for knockouts/technical knockouts, and 1.4 ± 2.1 for submissions, with an overall mean of 3.5 ± 4.3 across all groups [3]. A key contributor to post-training muscle soreness is eccentric muscle contractions. Research has shown that neuromuscular function can be impaired for several days following such exercises, largely due to muscle damage and the associated inflammatory response [4]. Therefore, interventions designed to alleviate the adverse effects of muscle damage and inflammation are recommended, including those that boost microcirculation, like sports massage or cooling [1,5]. The repeated, explosive movements inherent in MMA place significant mechanical and physiological demands on the neuromuscular system, leading to acute muscle damage, neuromuscular fatigue, and reduced performance, especially during periods of heavy training loads. Studies have indicated that repeated eccentric training can negatively affect how stiff muscles are, reduce leg power, lower pain tolerance, and impair balance, while also making exertion feel harder and delaying the recovery process [4,6].

In MMA training, the systematic organization of training periods reveals several factors impacting energy and muscle endurance, including the crucial aspect of regeneration [7]. Although a single standardized approach to recovery methods is lacking, a wide array of practical techniques is commonly utilized. These include the application of cold through water immersion [8] ice packs [9], the use of contrast therapy [5,10], various forms of massage such as dry massage [11,12], lymphatic drainage [1], instrument-assisted massage [13], and many other strategies. These methods offer tangible benefits beyond theoretical considerations, such as lessening the effects of delayed onset muscle soreness (DOMS) [9,14], improving the muscles’ ability to handle load [15], enhancing blood circulation within muscles [16], reducing inflammation [17] and alleviating muscle tension and stiffness [18], thereby positively affecting recovery and athletic output [1].

Existing research generally supports active recovery as superior to passive rest following exercise [19]; however, the impact of massage on stress, anxiety, and post-exercise regeneration remains inconclusive. While some studies indicate that massage can reduce stress and anxiety, improve recovery, and decrease inflammatory cytokines like TNF-α and IL-6, which increase after muscle damage [20], other research has shown no clear benefits from massage [21,22]. Additionally, massage has been suggested to stabilize muscle damage indicators such as creatine kinase (CK) and lactate dehydrogenase (LDH) levels, which may help protect muscle integrity after exercise [23]. However, the effectiveness of massage appears to be influenced by individual responses and the specific techniques employed [20], with some studies reporting mixed or inconclusive results on its impact on recovery [21,22]. Consequently, further research is necessary to refine massage protocols and determine their optimal use for different populations and exercise modalities.

Blood flow restriction (BFR) (a technique involving using a cuff or compression band to partially limit blood flow to the limbs) may enhance muscle recovery by promoting increased metabolic stress and cell swelling, which stimulate anabolic signaling pathways, such as mechanistic Target of Rapamycin (mTOR), and elevate the production of growth factors like Insulin-like Growth Factor 1 (IGF-1), ultimately improving muscle protein synthesis and reducing muscle damage [24,25]. Thus, BFR is emerging as a relatively new strategy for recovery [26]. Some evidence suggests that BFR might increase pressure pain thresholds (PPT), although the reported recovery times vary across studies. Notably, there is a current lack of research directly examining the effects of BFR training on reactive strength index (RSI), muscle stiffness (MS), total quality of recovery (TQR), or stability scores, indicating a gap in our understanding of its potential benefits in these specific areas [27]. Combining BFR with cooling represents an innovative approach to regeneration, but its effectiveness has not yet been confirmed through randomized clinical trials, to the best of our knowledge.

While individual benefits of various recovery techniques are known, a notable void exists in the scientific understanding of their combined impact, especially for MMA athletes who encounter distinct physical stressors and recovery hurdles. The central research question of this dissertation tackles the immediate effects of sports massage, BFR, and cold therapy on quadriceps recovery after eccentric exercise within this unique athletic population. Comparing massage alone with BFR + cold therapy (BFR/Cool) aims to assess whether a simpler, minimally invasive approach like manual massage could be surpassed by a more complex method like BFR + cold therapy, ultimately testing the potential cost–benefit relationship of each approach, particularly if one proves to be significantly more effective.

The study aims to quantitatively evaluate how these methods, when used together, influence muscle biomechanical properties, muscle pain, and muscle strength in relation to recovery duration following an integrated recovery approach. Gaining a thorough understanding of the synergistic effects of these interventions is crucial, as effective recovery strategies not only enhance performance but also provide evidence-based guidance for sports medicine and athlete management practices. Crucially, this research pioneers an investigation into the combined acute effects of massage, BFR, and cold therapy—a largely uncharted area, particularly within the high-demand context of MMA athlete recovery.

## 2. Materials and Methods

### 2.1. Study Design

**Figure 1:**
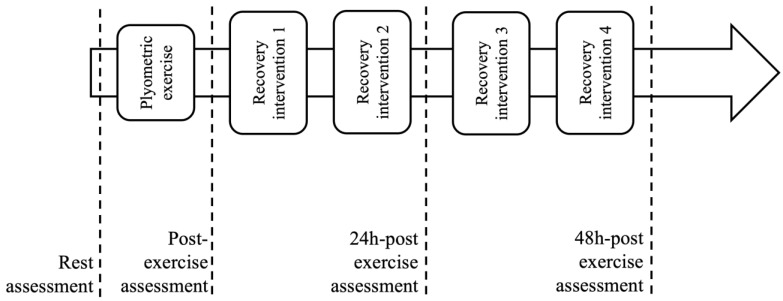
Study design.

A single-blind, randomized controlled trial investigated the impact of different recovery methods on 36 mixed martial arts (MMA) practitioners (Figure 1). The study involved 30 participants who were randomly assigned to one of three groups (n = 10 each): one group received massage therapy (massage), another underwent blood flow restriction training combined with cooling (BFR/cool), and the control group engaged in passive rest.

**Figure 2:**
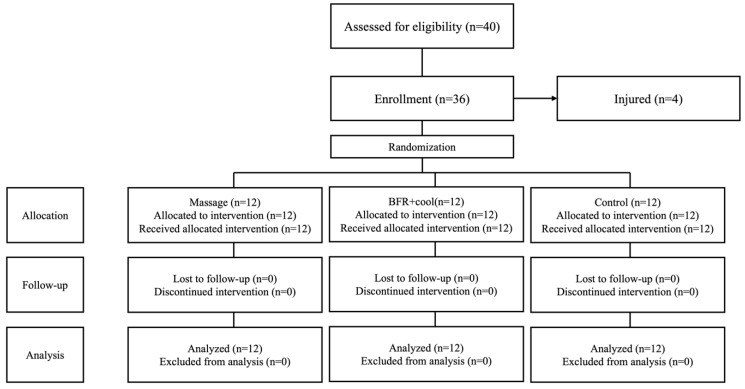
Participant flowchart.

All assessments were conducted between 9:00 and 11:00 a.m. at the Octagon MMA Sports Club in Żory, Poland. Prior to participation, all volunteers provided informed consent and completed a health questionnaire. Consistent environmental conditions (21 °C temperature, 50–55% humidity) were maintained throughout testing. A simple 1:1 randomization process, facilitated by randomizer.org, determined group assignment, independent of intervention duration and research staff. Furthermore, randomization dictated the session timing for the recovery intervention or passive rest (as shown in Figure 2). A familiarization session, which included two sets of 30-s plyometric jumps with massage and BFR+ice cooling between sets, was conducted seven days before the main study. Ethical approval for this research was obtained from the Polish Physiotherapy Society (no. 3/03/2024), and the study was registered in the clinical trials registry (doi.org/10.1186/ISRCTN15418049), adhering to the principles of the Declaration of Helsinki.

### 2.2. Participants

The participant pool for this investigation consisted of 36 mixed martial arts practitioners (28 males, 8 females) exhibiting a mean age of 28 ± 4 years, a body mass index of 25.0 ± 2.3 kg/m^2^, and an average of 11 ± 4 years of training in combat sports (as detailed in Table 1 and Table 2). Inclusion in the study required participants to be aged 18–40, possess at least three years of combat sports training, and train a minimum of five times weekly. Based on McKay’s classification, these athletes were categorized as level 3, indicating a highly trained or national level of expertise [28]. Furthermore, participants reported engaging in motor training sessions, which incorporated plyometric exercises, at least once per week. Exclusion from the study involved several factors: pre-study elevated blood pressure (above 140/90 mm Hg), current injuries, compromised skin integrity or unspecified skin lesions at measurement locations, the presence of tattoos at measurement sites (due to potential interference with tissue perfusion measurements), and the use of any medications, including analgesics and hormonal treatments. Individuals experiencing extreme fatigue, fever, or infection, or those who explicitly requested withdrawal at any point were also excluded. All participants provided written informed consent after receiving comprehensive information about the study procedures. To ensure accurate tissue perfusion measurements, participants were instructed to abstain from consuming any ergogenic aids (a list of prohibited substances was provided) for a four-hour period preceding the study.

The data analysis from Table 1 did not reveal any statistically significant differences (*p* < 0.05) between the groups concerning the examined variables.

According to the analysis of the results presented in Table 2, the distribution of participant sex was not significantly different across the various groups (*p* > 0.05).

### 2.3. Muscle Fatigue Protocol

Participants in the study performed familiar plyometric jumps onto a 50 cm high box [5]. Prior to the study, these MMA fighters confirmed their ability to execute this exercise, which they regularly incorporated into their training (at least weekly). The fatigue protocol involved five sets of these plyometric jumps, continuing until the participant could no longer maintain the effort, with a one-minute rest period between each set to simulate aspects of an MMA bout. Throughout the fatigue protocol, the total number of jumps accumulated over the five sets averaged 77.2 ± 5.6 in the massage group, 79.3 ± 4.8 in the BFR/cooling group, and 78.3 ± 6.2 in the control group. Before this fatigue task, participants completed a 5-min moderate-intensity cycling warm-up and a 3-min stretching routine targeting leg muscles (including knee flexors and extensors, hip abductors and adductors, and triceps surae). Throughout the jump protocol, a paramedic and an assistant monitored participants, ensuring correct jump execution. The use of box jumps is often recommended in MMA training due to their potential to reduce ground reaction forces upon landing, thereby mitigating the elevated injury risk typically associated with high eccentric loads [10]. Each box jump comprised distinct phases: 1. Starting position: standing before the box, lowering the body by bending hips and knees while dorsiflexing ankles and extending arms backward; 2. Concentric phase: a powerful upward and forward jump, extending hips, knees, and ankles (plantarflexion), while swinging arms upward; 3. Eccentric phase: absorbing the landing on the box by flexing hips, knees, and ankles to dissipate force [10]. Throughout the repetitions, the athletes, who were already experienced in plyometric training, were clearly instructed and motivated to be as explosive as possible and jump as high as possible, ensuring maximal intent in all repetitions [29].

### 2.4. Recovery Intervention

The study participants were randomly allocated into three distinct recovery groups. The massage group underwent massage therapy, employing techniques such as effleurage, petrissage, friction, and vibration, following established protocols in scientific literature. Each thigh received a 10-min massage, totaling 20 min of massage overall [30,31]. The BFR/cool group experienced a combined intervention of blood flow restriction and cooling. This involved the application of thigh compression cuffs, which participants inflated to 200 mmHg, equivalent to 100% arterial occlusion pressure (AOP). This occlusion was maintained for two minutes, after which ice packs were applied to the entire surface area of the quadriceps femoris muscle for eight minutes. This cycle of BFR followed by cooling was repeated for a total of 20 min. The control group served as the passive control, receiving no active intervention and engaging solely in passive rest. For each participant, two experienced sports physiotherapists (each with at least three years of experience) were randomly assigned to oversee and administer their recovery treatments. A total of four recovery sessions were conducted for each participant, occurring twice daily, once in the morning (9:00–11:00) and once in the evening (18:00–20:00). The athletes were instructed to avoid taking any nutritional supplements or drugs during the 48-h period following exercise and to maintain their usual dietary habits.

### 2.5. Outcomes

To identify the midpoint of the rectus femoris muscle’s length in both legs, ultrasound imaging (using a Sonoscope E2 from China) was employed to visualize the muscle’s origin at the anterior inferior iliac spine and its insertion at the tibial tuberosity. The muscle length was measured as the distance between these two landmarks, and the midpoint was calculated by dividing this distance in half. This location was then marked on the skin. The study’s measurements encompassed transcutaneous hyperemia (in conventional perfusion units), elasticity (in relative arbitrary units), pressure pain threshold (in Newtons per centimeter squared), reactive strength index (in meters per second squared), and total quality recovery (on a scale of 0 to 20). These measurements were taken under standardized conditions and positions, as outlined in relevant scientific literature, at five distinct time points: (i) prior to exercise, (ii) immediately after exercise, (iii) 24 h post-exercise—specifically 5 min after the second recovery session, (iv) 48 h post-exercise—specifically 5 min after the fourth recovery session.

#### 2.5.1. Tissue Perfusion (PU)

To evaluate microcirculation within living tissue, the study employed Laser Doppler flowmetry (LDF) using a Perimed PeriFlux 6000 Combined System (Stockholm, Sweden). Blood flow assessment involved placing a probe at the designated skin location, following established guidelines [32] with a measurement depth of 2.5 mm and a volume of 1 mm³, over a two-minute period. LDF, recognized for its consistent results, sensitivity, and non-invasive nature, allows for precise measurement of microcirculatory function both at rest and during physiological challenges [33]. Often considered the most reliable technique for assessing microcirculation, LDF provides exceptional accuracy and consistency [34], and the resulting blood flow data are quantified in perfusion units. Evaluation was performed with three repetitions per assessment, and the average was used as the result.

#### 2.5.2. Biomechanical Properties—Elasticity (E [arb: Relative Arbitrary Unit])

The biomechanical characteristics of the rectus femoris muscle (specifically its tone, stiffness, and elasticity) were evaluated using a MyotonPRO device (Myoton, Estonia, 2021). This digital palpation instrument comprises a main unit and a depth probe with a diameter of 3 mm [35]. The MyotonPRO operates by employing a dynamic mechanical response technique. This involves delivering a precisely controlled mechanical tap to the tissue and recording its subsequent dynamic behavior, specifically displacement and acceleration. From these recordings, the device’s internal algorithms calculate parameters that define the biomechanical properties under investigation [36]. The scientific literature supports the reliability and consistency of this device [37]. During measurement, the probe is applied perpendicularly to the muscle with an initial force of approximately 0.18 N. The device then emits a brief mechanical impulse (0.4 N for 15 milliseconds), causing temporary tissue deformation. By analyzing the resulting tissue vibrations, the extent of deformation, the recovery time to the original state, and the damping of these vibrations, the MyotonPRO determines relative values for specific indicators of muscle stiffness, tension, and elasticity [38]. Evaluation was performed with three repetitions per assessment, and the average was used as the result.

#### 2.5.3. Pressure Pain Threshold (PPT [N/cm])

The pressure pain threshold (PPT) was quantified using an FPIX algometer (Wagner Instruments, Greenwich, CT, USA, 2013). This measurement aimed to provide an objective assessment of pain sensitivity [39]. Following the manufacturer’s guidelines, PPT was assessed on the rectus femoris muscle (RF). Each participant underwent three applications of a pressure probe (with a radius of 4 mm) over a consistently marked area, inducing compressive forces. Evaluation was performed with three repetitions per assessment, and the average was used as the result. The resulting force value, expressed in Newtons per centimeter squared (N/cm^2^), was digitally displayed and calculated as the mean of the three repetitions. Pressure application continued until the participant indicated that the sensation became unpleasant [5].

#### 2.5.4. Reactive Strength Index (RSI[m·s^−1^])

Reactive strength index (RSI), an indicator of an individual’s capacity for rapid transitions between eccentric and concentric muscle actions and a measure of reactive strength, was assessed using a Force Decks ground reaction force plate (Vald-Performance, Australia, 2012), a device known for its high reliability and repeatability [40]. To measure RSI, participants were instructed to jump off a 50 cm box and immediately perform a maximal vertical jump upon landing on the force plate [10]. Prior to each RSI measurement (except after the fatigue protocol), a 5-min stationary cycling and 3-min leg stretching warm-up was conducted. A research assistant provided instructions on the jumping technique, and participants completed a familiarization trial. Then, from a total of three trials, interspaced by 30-s, the best result was selected for further analysis [41]. RSI was calculated using the formula: Jump height / Contact time (measured in meters per second squared) [42].

#### 2.5.5. The Total Quality of Recovery (TQR)

Total Quality Recovery (TQR) serves as a framework for evaluating and tracking athlete recovery, encompassing both physiological and psychological aspects. This adapted scale assesses the quality of an athlete’s recovery throughout training periods, offering insights into how well healthy individuals recuperate from physical exertion [43]. To optimize training adaptations and prevent issues like overuse injuries or non-contact injuries, coaches in competitive sports should consistently monitor recovery status before and after key training sessions or competitions, thereby supporting overall well-being. Consequently, the systematic quantification and assessment of an athlete’s psychological state of recovery during a season, utilizing reliable subjective measures such as the TQR scale, is crucial [44]. Since the TQR scale reflects an individual’s overall perception of recovery throughout the day, only a single measurement was taken at rest on Day 1. A post-exercise measurement was not collected, as it would not allow sufficient time for participants to fully perceive their recovery status. Additionally, it could have been influenced by the immediate perception of exercise intensity, potentially compromising the accuracy of the results.

### 2.6. Statistical Methods

Statistica v.13.1 (StatSoft Polska, Kraków) was the software used for all analyses. Descriptive statistics (means, standard deviations, 95% confidence intervals) were calculated. Assumptions of normality, homogeneity of variance, and sphericity were checked using Shapiro–Wilk, Levene, and Mauchly tests. A repeated-measures MANOVA was used to assess significance, with effect sizes (η^2^) classified as small (0.01–0.059), moderate (0.06–0.137), or large (>0.137). Significant MANOVA results were followed up with Bonferroni post hoc tests. Nonparametric Friedman and Kruskal–Wallis tests were used if normality was violated. Statistical significance was set at *p* < 0.05. A prior power analysis (G*Power) indicated a required minimum sample of 33 participants for a repeated-measures ANOVA (within-between interaction, effect size ≥ 0.3, α = 0.05, 1 − β = 0.95) to achieve a power of 0.96.

## 3. Results

Table 3 presents the basic descriptive statistics and corresponding 95% confidence intervals for the analyzed groups. It also shows the results of either the parametric or nonparametric repeated-measures analysis of variance

**Figure 3:**
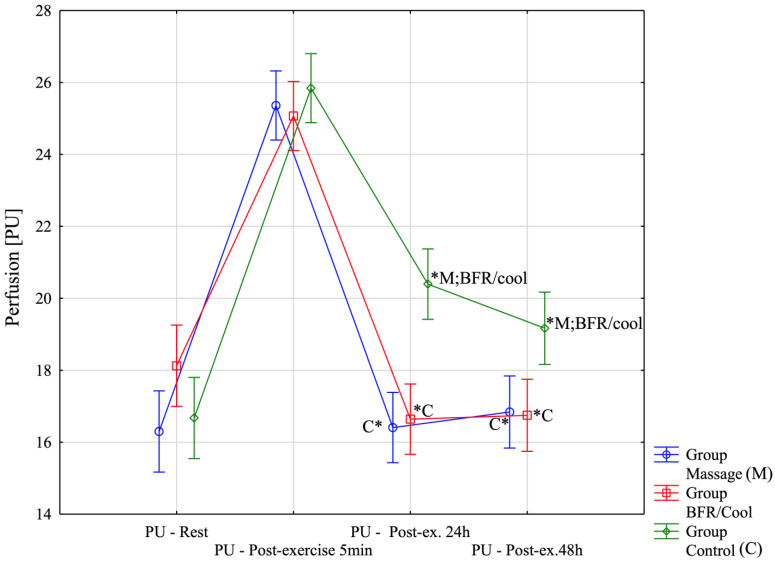
Changes in perfusion unit (PU) across the four assessment time points for all three groups. *: significantly different from other group (*p* < 0.05).

The analysis of the results presented in Figure 3 began with interpreting the variable PU. Due to the lack of distribution normality, a nonparametric Friedman analysis of variance and multiple-comparison tests were applied for comparisons between repeated measures within the analyzed groups. In all groups, significant differences were observed for the following comparisons: rest–post-exercise 5 min, post-exercise 24 h, and post-exercise 48 h. Specifically, the results for massage yielded χ^2^ = 23.50, *p* = 0.00003; for BFR/Cool; χ^2^ = 24.70, *p* = 0.00002; and for the control group, χ^2^ = 28.00, *p* < 0.00001. In each group, significant differences were found between rest–post-exercise 5 min (*p* < 0.0001), post-exercise 5 min–post-exercise 24 h (the second recovery session; *p* < 0.0001), and post-exercise 5 min–post-exercise 48 h (the fourth recovery session; *p* < 0.0001). A nonparametric Kruskal–Wallis analysis of variance and multiple-comparison tests were employed to verify differences in PU between groups. No significant differences were noted between groups at rest (H(2, n = 36) = 4.49; *p* = 0.11), nor immediately following the experiment (post-exercise 5 min; H(2, n = 36) = 1.85; *p* = 0.39). However, significant differences were observed between groups at post-exercise 24 h (H(2, n = 36) = 17.62; *p* = 0.0001). Results in the massage group (*p* = 0.0004) and BFR/cool group (*p* = 0.018) were significantly lower (i.e., better) than in the control group, although no significant differences were detected between the massage and BFR/cool groups (*p* = 0.99). Similarly, significant differences between groups were found at post-exercise 48 h (H(2, n = 36) = 10.01; *p* = 0.0067). Results in the massage group (*p* = 0.016) and the BFR/cool group (*p* = 0.023) were again significantly lower (better) than in the control group, with no significant difference detected between the massage and BFR/cool groups (*p* = 0.99).

**Figure 4:**
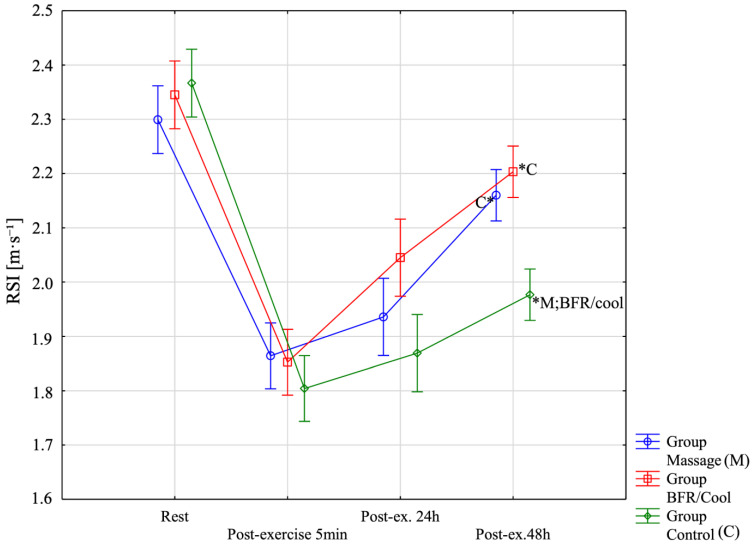
Reactive strength index (RSI) across the four assessment time points for all three groups. *: significantly different from other group (*p* < 0.05).

To assess the statistical significance of differences in the RSI [m⋅s^−1^] variable, a repeated-measures analysis of variance was conducted (Figure 4). Significant main effects were observed for Group F = 6.78; *p* = 0.0034; η^2^ = 0.29 and Before–After F = 223.27; *p* < 0.0001; η^2^ = 0.87, as well as for the Group times× Before–After interaction F = 6.90; *p* < 0.0001; η^2^ = 0.30. In the BFR/cool group, all pairwise comparisons between time points were statistically significant (*p* < 0.001). In the massage group, the only nonsignificant comparison was between post-exercise 5 min and post-exercise 24 h (*p* = 0.99); all other comparisons were significant (*p* < 0.01). In the control group, no significant differences were found between post-exercise 5 min and post-exercise 24 h (*p* = 0.99) or between post-exercise 24 h and post-exercise 48 h (*p* = 0.20). All other pairwise comparisons in the control group were significant (*p* < 0.001). For RSI Post-exercise 48 h, results were significantly higher (i.e., better) in the massage group (*p* = 0.002) and the BFR/cool group (*p* < 0.0001) compared with the control group. These findings are also illustrated in the figure below.

**Figure 5:**
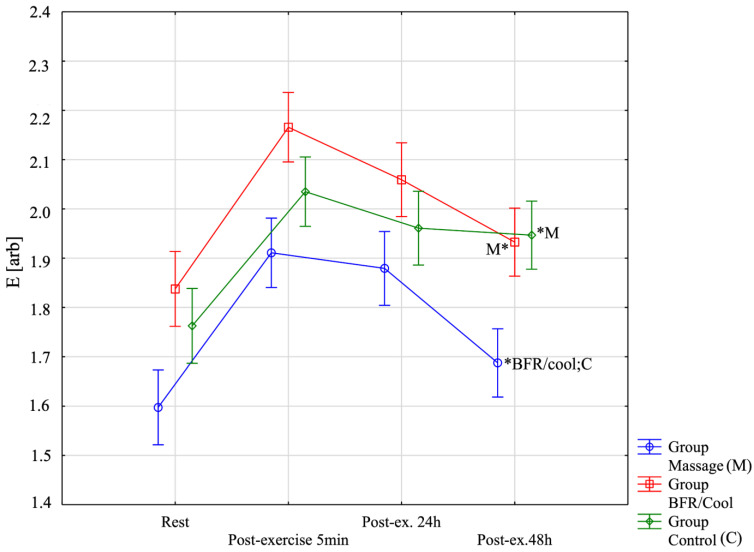
Muscle elasticity (E) across the four assessment time points for all three groups. *: significantly different from other group (*p* < 0.05).

To verify the significance of differences in Elasticity E [arb] (Figure 5), a repeated-measures analysis of variance was performed. Significant main effects were observed for Group F = 13.24; *p* = 0.0001; η^2^ = 0.45, Before–After F = 171.92; *p* < 0.0001; η^2^ = 0.84, and the Group × Before–After interaction F = 5.79; *p* < 0.0001; η^2^ = 0.26. Within the BFR/Cool group, all pairwise comparisons among time points were statistically significant (*p* < 0.05). In the massage group, the only nonsignificant difference was between post-exercise 5 min and post-exercise 24 h (*p* = 0.99); all other comparisons were significant (*p* < 0.05). In the control group, no significant differences were identified between post-exercise 5 min and post-exercise 24 h (*p* = 0.24) nor between post-exercise 24 h and post-exercise 48 h (*p* = 0.99). All other comparisons in the control group were significant (*p* < 0.001). At post-exercise 48 h [4th session], E [arb] values in the massage group were significantly lower (i.e., better) than in both the BFR/cool group (*p* = 0.001) and the control group (*p* < 0.0001). This finding is also illustrated in the figure below.

**Figure 6:**
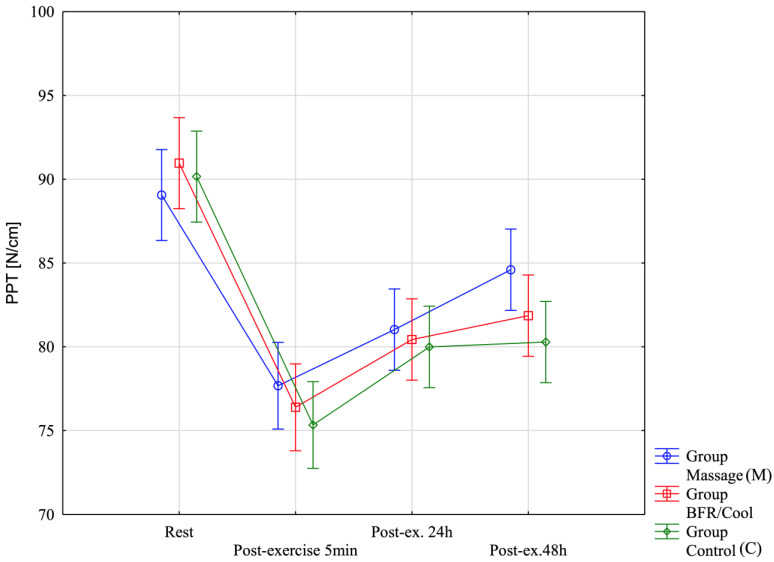
Pressure pain threshold (PPT) across the four assessment time points for all three groups.

To verify the significance of differences for the PPT [N/cm] variable (Figure 6), a repeated-measures analysis of variance was applied. Significant differences were observed only for the main effect of Before–After: =63.67; *p* < 0.0001; η^2^ = 0.66. No significant differences were observed for the main effect of Group (F = 1.67; *p* = 0.20; η^2^ = 0.091) or the interaction (F = 1.00; *p* = 0.43; η^2^ = 0.057). This finding is also illustrated in the figure below.

**Figure 7:**
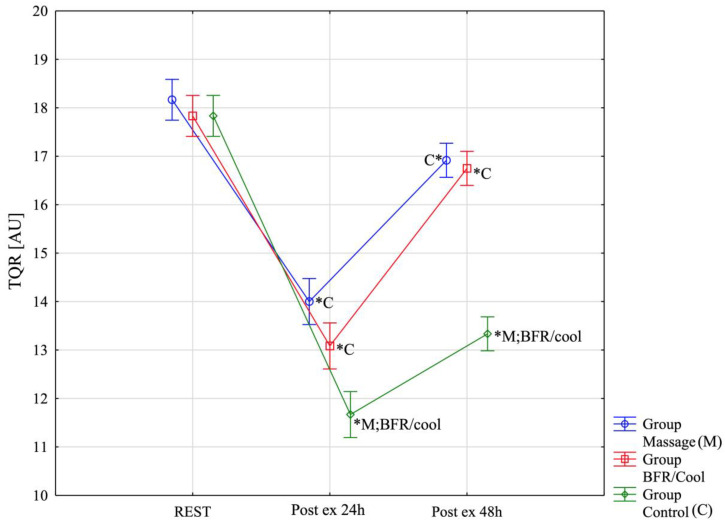
Total quality recovery (TQR) across the assessment time points for all three groups. *: significantly different from other group (*p* < 0.05).

Due to the lack of normal distribution, the nonparametric Friedman analysis of variance and multiple-comparison tests were used for the TQR variable (Figure 7). Analyses in all groups indicated significant differences between rest/post-exercise 5 min, post-exercise 24 h, and post-exercise 48 h: Massage: χ^2^ = 23.13, *p* = 0.00001; BFR/cool: χ^2^ = 23.13, *p* = 0.00001; Control: χ^2^ = 23.53, *p* = 0.00001. In all groups, significant differences were observed between rest and 24 h (*p* < 0.0001), as well as between 24 h and post-exercise 48 h (4th session) (*p* < 0.0001). To verify the significance of differences for the variable PU between groups, a nonparametric Kruskal–Wallis analysis of variance and multiple-comparison tests were conducted. No significant differences were found among the groups at rest (H(2, n = 36) = 1.73; *p* = 0.42). However, significant differences were observed in rest/post exercise (H(2, n = 36) = 21.92; *p* < 0.0001). Specifically, both the massage group (*p* < 0.0001) and the BFR/cool group (*p* = 0.041) scored significantly higher (better) than the control group, with no significant differences noted between the massage and BFR/cool groups (*p* = 0.99). Significant differences between groups were also found at post-exercise 48 h (H(2, n = 36) = 27.19; *p* < 0.0001). The massage group (*p* < 0.0001) and the BFR/cool group (*p* = 0.0002) again scored significantly higher (better) than the control group. No significant differences were observed between massage and BFR/cool (*p* = 0.99).

## 4. Discussion

The study showed that both sports massage and a combination of blood flow restriction (BFR) with cold therapy significantly improved recovery outcomes in MMA athletes following fatigue. Specifically, both interventions led to superior recovery of PU, elasticity (E), RSI, and TQR at 24 and 48 h post-exercise, compared to the control group. Statistical analyses showed significant main effects and interaction effects for time and group in variables such as RSI, elasticity, and PU. Both experimental groups consistently outperformed the control group, particularly at the 48-h mark, while no significant differences were found between the massage and BFR/cool groups. However, no significant group differences were observed for PPT, despite a significant improvement over time in all groups. These findings suggest that both interventions are effective strategies to accelerate muscle recovery in combat sports athletes.

The analysis revealed significant within-group differences in PU at multiple recovery time points (post-exercise 5 min, 24 h, and 48 h) across all groups, with better recovery outcomes observed in the massage and BFR/cool groups compared to the control group at 24 and 48 h post-exercise. However, no significant differences were found between the massage and BFR/cool groups at any time point, suggesting similar efficacy between these two interventions. Massages are known to enhance blood flow [45], reduce muscle stiffness [46], and facilitate the clearance of metabolic byproducts [14], which can collectively reduce perceived discomfort and improve muscle recovery. Similarly, BFR combined with cooling may induce a hormetic response that enhances cellular repair mechanisms [47], reduces inflammation, and limits muscle damage by constraining perfusion [25] while applying a cooling stimulus. The cooling component may also attenuate nerve conduction velocity and reduce the perception of pain [48], while BFR may promote anabolic signaling pathways and muscle regeneration [49].

The results indicate that both time and intervention significantly influenced muscle elasticity following exercise, with massage emerging as the most effective strategy for restoring muscle mechanical properties by 48 h post-exercise. This finding aligns with prior evidence suggesting that massage facilitates recovery by promoting intramuscular fluid exchange, reducing edema, and modulating the viscoelastic properties of muscle tissue through mechanical and neurological pathways [11]. The significantly lower elasticity values observed in the massage group compared to the BFR/cool and control groups may reflect these mechanisms, as massages likely reduce muscle tone and stiffness more effectively than other modalities. In contrast, the combined BFR/cool intervention may have produced competing physiological effects—hypoxia from BFR and reduced tissue temperature from cooling—that did not support an optimal recovery of muscle elasticity [50]. The control group’s slower recovery further supports the role of active interventions in mitigating the typical post-exercise increases in stiffness associated with delayed-onset muscle soreness [4,12,51].

In contrast, changes in PPT appeared to be primarily time-dependent, with no significant differences observed between groups or for the interaction effect. This suggests that pain sensitivity improved naturally over the 48-h period, regardless of intervention, consistent with the known time course of DOMS recovery. The absence of group differences in PPT may reflect the complexity of nociceptive mechanisms involved in pain perception, which are not always directly influenced by interventions that target mechanical recovery [52]. It also highlights that while massages may accelerate the restoration of muscle mechanical properties, their effects on sensory recovery may be less pronounced or more variable among individuals.

Results regarding RSI revealed significant main effects and interactions, indicating that both time and group influenced performance recovery, with the BFR/cool and massage groups showing greater improvements than the control group. At 48 h post-exercise, RSI values were significantly higher in both intervention groups compared to control, suggesting enhanced neuromuscular recovery from the applied recovery strategies. The enhancement in RSI seen in the BFR/cool group may be explained by the post-exercise application of blood flow restriction, which has been shown to stimulate anabolic signaling pathways (e.g., mTOR) and promote faster recovery of muscle function by increasing systemic growth hormone levels and cellular swelling [47]. Cooling, when combined with BFR, may have attenuated the inflammatory response and reduced muscle temperature, helping to preserve neuromuscular efficiency without significantly impairing muscle contractility [25]. Similarly, massages have been found to improve muscle performance possibly by enhancing circulation, and decreasing neuromuscular inhibition, which may contribute to the observed RSI improvements [53].

TQR scores significantly changed over time in all groups, with marked differences between rest, 24 h, and 48 h post-exercise, indicating progressive recovery regardless of intervention. TQR ratings post-exercise and at 48 h were significantly higher in both the massage and BFR/cool groups compared to control, suggesting that these interventions were perceived as more effective, though no differences were found between the two active recovery methods. This aligns with literature showing that recovery interventions, particularly massage and modalities involving compression or cooling, enhance psychological perceptions of recovery, likely due to their immediate soothing effects and association with professional care [51]. The similar TQR scores between massage and BFR/cool suggest both interventions were equally well-received, reinforcing the value of combining physiological recovery strategies with interventions that also address athlete perceptions and psychological readiness.

Despite the findings of this study, several limitations should be acknowledged. First, the sample size was relatively small and limited to mixed martial arts athletes, which may affect the generalizability of the results to other athletic populations or sports disciplines. Moreover, it would be important to include an active control group in addition to the passive one to determine whether such a group would influence the comparisons. Additionally, in a more complex scenario, it would be interesting to compare massages alone, massages with cold therapy, BFR alone, BFR + cold therapy, and cold therapy alone. This would create more comparable conditions and help isolate which approach provides the best results. However, such an expansion could increase the complexity of implementation, particularly in experimental studies with athletes, as their training routines could be impacted. Additionally, a cross-sectional design is recommended to reduce potential heterogeneity associated with acute effects and to help identify whether individuals respond differently to a given modality. Also, although efforts were made to standardize the intervention protocols, individual variability in response to massage and BFR/cold therapy may have influenced the outcomes. The study also relied on short-term follow-up (up to 48 h), which does not provide insight into the long-term effects or potential cumulative benefits of repeated application of these recovery strategies. Another limitation is the lack of biochemical markers such as creatine kinase or inflammatory cytokines, which could have provided deeper insights into the physiological mechanisms underpinning recovery. Furthermore, the subjective nature of some assessments, such as TQR, may introduce response bias. Future research should explore larger and more diverse athlete populations, include longer monitoring periods, and integrate objective biochemical and imaging markers. Additionally, further studies should investigate the optimal timing, duration, and frequency of combined BFR and cold therapy, as well as compare these approaches with other emerging recovery modalities to better tailor recovery strategies to individual athlete needs and training contexts.

## 5. Conclusions

The study highlighted the contribution of acute recovery interventions, specifically sports massage combined with blood flow restriction and ice, to muscle recovery following eccentric exercise in mixed martial arts athletes. By focusing on these combined modalities, the study sought to address everyday performance and injury issues faced by athletes participating in high-intensity sports. Results revealed reduced muscle soreness and improved strength loss due to acute fatigue in participants who received the integrated treatment compared to the control group, thus providing a solid approach to accelerated recovery. This study thus effectively addressed the initial research question by establishing a clear association between combined recovery techniques and improved muscle recovery outcomes, offering empirical support for integrating these interventions into sports training programs. The practical implications emphasize the need to incorporate multimodal recovery strategies to optimize athletic performance and enhance recovery protocols, which may be particularly important when designing training programs that prioritize athlete health and sustainability.

## Figures and Tables

**Table 1:** Characteristics of the study sample by group.

	Group n= 36		
	Massage n = 12	BFR/Cool n = 12	Control n = 12
	M ± SD		
Age	28 ± 3	29 ± 4	28 ± 5
Height [cm]	176.3 ± 11.4	175.8 ± 10.1	175.6 ± 10.4
Weight [kg]	77.8 ± 12.6	78.1 ± 16.1	79.0 ± 17.0
Training experience (years)	10 ± 2	11 ± 4	12 ± 5
Body mass index (kg/m^2^)	24.40 ± 1.73	24.99 ± 2.33	25.53 ± 2.66

**Table 2:** Characteristics of the study sample by group and sex.

Sex	Group			Totals
	Massage	BFR/Cool	Control	
m	10	9	9	28
%	83.33%	75.00%	75.00%	
f	2	3	3	8
%	16.67%	25.00%	25.00%	
summary	12	12	12	36
χ^2^ = 0.33; df = 2; *p* = 0.85				

BFR: blood flow restriction.

**Table 3:** Basic descriptive statistics, 95% CIs, and parametric/nonparametric repeated-measures ANOVA results (n = 36).

Variables	Group			Main Effects and Interaction for MANOVA Repeated Measures F/*p*/η^2^ Or χ^2^; *p*
	Massage (n = 12)	BFR/Cool (n = 12)	Control (n = 12)	
	M ± SD (95%;95%CI)	M ± SD (95%;95%CI)	M ± SD (95%;95%CI)	
PU-Rest	16.30 ± 2.28 (14.85;17.75)	18.13 ± 1.92 (16.90;19.35)	16.68 ± 1.47 (15.74;17.61)	Massage: χ^2^ χ^2^ = 23.50; *p* = 0.00003 BFR/Cool χ^2^ = 24.70; *p* = 0.00002 Control χ^2^ = 28.00; *p* < 0.00001
PU-Post-exercise 5 min	25.36 ± 2.04 (24.06;26.65)	25.07 ± 1.43 (24.16;25.97)	25.84 ± 1.35 (24.98;26.70)	
PU-Post-ex. 24 h [2 session recovery]	16.41 ± 1.76 (15.29;17.52)	16.64 ± 1.44 (15.72;17.56)	20.39 ± 1.77 (19.27;21.52)	
PU-Post-ex. 48 h [4 session]	16.84 ± 1.91 (15.63;18.06)	16.75 ± 1.32 (15.91;17.59)	19.17 ± 1.83 (18.00;20.33)	
RSI [m s^−1^]-Rest	2.30 ± 0.11 (2.23;2.37)	2.35 ± 0.11 (2.28;2.41)	2.37 ± 0.10 (2.30;2.43)	Group: F = 6.78; *p* = 0.0034; η^2^ = 0.29 Before-After: F = 223.27; *p* < 0.0001; η^2^ = 0.87 Group x Before-After: F = 6.90; *p* < 0.0001; η^2^ = 0.30
RSI [m s^−1^]-Post-exercise 5 min	1.86 ± 0.14 (1.77;1.95)	1.85 ± 0.04 (1.82;1.88)	1.80 ± 0.10 (1.74;1.87)	
RSI [m s^−1^]-Post-ex. 24 h [2 session recovery]	1.94 ± 0.13 (1.85;2.02)	2.05 ± 0.06 (2.00;2.09)	1.87 ± 0.15 (1.77;1.97)	
RSI [m s^−1^]-Post-ex. 48 h [4 session]	2.16 ± 0.11 (2.09;2.23	2.20 ± 0.07 (2.16;2.24)	1.98 ± 0.05 (1.94;2.01)	
E [arb]-Rest	1.60 ± 0.12 (1.52;1.67)	1.84 ± 0.17 (1.73;1.94)	1.76 ± 0.09 (1.71;1.82)	Group: F = 13.24; *p* = 0.0001; η^2^ = 0.45 Before-After: F = 171.92; *p* < 0.0001; η^2^ = 0.84 Group x Before-After: F = 5.79; *p* < 0.0001; η^2^ = 0.26
E [arb]-Post-exercise 5 min	1.91 ± 0.12 (1.84;1.98)	2.17 ± 0.14 (2.08;2.25)	2.04 ± 0.11 (1.97;2.10)	
E [arb]-Post-ex. 24 h [2 session recovery]	1.88 ± 0.12 (1.80;1.96)	2.06 ± 0.13 (1.98;2.14)	1.96 ± 0.13 (1.88;2.04)	
E [arb]-Post-ex. 48 h [4 session]	1.69 ± 0,14 (1.60; 1.78)	1.93 ± 0.11 (1.86; 2.00)	1.95 ± 0.10 (1.88;2.01)	
PPT [N/cm]-Rest	89.06 ± 5.77 (85.39;92.72)	90.96 ± 4.03 (88.40;93.52)	90.16 ± 3.82 (87.73;92.58)	Group: F = 1.67; *p* = 0.20; η^2^ = 0.091 Before-After: F = 63.67; *p* < 0.0001; η^2^ = 0.66 Group x Before-After: F = 1.00; *p* = 0.43; η^2^ = 0.057
PPT [N/cm]-Post-exercise 5 min	77.68 ± 5.56 (74.14;81.21)	76.39 ± 2.73 (74.65;78.13)	75.33 ± 4.45 (72.51;78.16)	
PPT [N/cm]-Post-ex. 24 h [2 session recovery]	81.03 ± 3.37 (78.88;83.17)	80.43 ± 4.16 (77.79;83.08)	79.99 ± 4.75 (76.98;83.01)	
PPT [N/cm]-Post-ex. 48 h [4 session]	84.60 ± 3.41 (82.43;86.77)	81.86 ± 3.80 (79.44;84.28)	80.28 ± 5.01 (77.10;83.47)	
TQR-REST	18.17 ± 0.72 (17.71;18.62)	17.83 ± 0.72 (17.38;18.29)	17.83 ± 0.72 (17.38;18.29)	Massage: χ^2^ χ^2^ = 23.13; *p* < 0.0001 BFR/Cool χ^2^ = 23.13; *p* < 0.0001 Control χ^2^ = 23.53; *p* < 0.0001
TQR-Post ex-5 min	14.00 ± 0.85 (13.46;14.54)	13.08 ± 0.51 (12.76;13.41)	11.67 ± 0.98 (11.04;12.29)	
TQR-Post ex, 48 h	16.92 ± 0.51 (16.59;17.24)	16.75 ± 0.45 (16.46;17.04)	13.33 ± 0.78 (12.84;13.83)	

PU: perfusion unit; PPT: pressure pain threshold; RSI: reactive strength index; TQR: total quality recovery; E: elasticity: Post ex: post-exercise.

## Data Availability

The data presented in this study are available on request from the corresponding author. The data are not publicly available due to data protection.

## Abbreviations

The following abbreviations are used in this manuscript:

TQR	Total quality recovery
PPT	Pressure pain threshold
BFR	Blood flow restriction
RSI	Reactive strength index
